# Titanium dioxide in dental enamel as a trace element and its variation with bleaching

**DOI:** 10.4317/jced.54478

**Published:** 2018-06-01

**Authors:** Tatiana Vargas-Koudriavtsev, Randall Durán-Sedó, Óscar-Andrey Herrera-Sancho

**Affiliations:** 1 Dental Faculty, University of Costa Rica. Postgraduate Program in Prosthodontics, University of Costa Rica; 2 Dental Faculty, University of Costa Rica. Graduate program dental student; 3 School of Physics, University of Costa Rica. Materials Research Science and Engineering Center, University of Costa Rica. Institute for Quantum Optics and Quantum Information, Austrian Academy of Sciences, Innsbruck, Austria

## Abstract

**Background:**

Titanium is a less studied trace element in dental enamel. Literature relates an increased Titanium concentration with a decreased enamel crystal domain size, which in turn is related to a higher color value. The aim of our study was to analyze the effect of tooth bleaching agents on its concentration in dental enamel by means of confocal Raman spectroscopy.

**Material and Methods:**

Human teeth were randomly distributed in six experimental groups (n=10) and submitted to different bleaching protocols according to the manufacturer´s instructions. Confocal Raman spectroscopy was carried out in order to identify and quantify the presence of titanium dioxide molecules in enamel prior to and during whitening. Statistical analysis included repeated measures analysis of variance (*p*≤0.05) and Bonferroni pairwise comparisons.

**Results:**

Titanium dioxide concentration was negatively affected by the longer bleaching protocols (at-home bleaching gels). All in-office whitening products increased significantly the studied molecule (*p*≤0,05).

**Conclusions:**

All dental specimens depicted the presence of titanium dioxide as a trace element in dental enamel. Bleaching gels that have to be applied at higher concentrations but for shorter periods of time increase the concentration of titanium dioxide, whilst at-home whitening gels used for longer periods of time despite the lower concentration caused a loss in titanium.

** Key words:**Bleaching, whitening, hydrogen peroxide, carbamide peroxide, Raman spectroscopy, titanium dioxide.

## Introduction

Dental enamel consists primarily of hydroxyapatite (HA), which is composed of calcium, phosphate and hydroxyl ions. However, there are other substances present in very small amounts called “trace materials”, which might be related to enamel physical properties. Among these substances, titanium can be found, which is a less studied trace element related to enamel color and hardness.

To confirm the presence of these materials, since the early 1930s, Drea obtained spectra of 18 dried teeth by means of exciting the trace elements with a direct current method, and found out that there are several elements present in human enamel and dentin. Among those can be mentioned: Al, Fe ,Ba, Cu, F, Pb, Li, Mn, K, Si, Ag, Sr, V, Zn and Ti. (1) According to some authors, the content of trace materials is related to the subject´s dietary and life style characteristics (2), other researches who have studied enamel trace materials in animals believe they are related to geographic location, and that these are key elements for complex archeological and biology studies, among others (3).

Some investigators relate the content of trace elements with the presence of caries. Riyat and Sharma observed that carious teeth presented a slight decrease in elements such as Sr, Ca, Mg, P, Be, V, Ni, Zn, Nb, Ag, Cd, Sb, Ba, La, W, Pb, Bi and Ti (4). Their results are complementary with another study whose authors suggest that Zn is present in a population less prone to caries formation (5). The results of these studies are not conclusive and often contradictory, which shows that further research is necessary in order to understand the role of trace elements in the caries formation.

Other investigations relate trace elements with the crystallographic characteristics of HA, whose size might be eventually related to its optical and physical properties. Eimar and others found that a smaller size of the enamel crystals was the major predictor for higher tooth lightness (6), they also found that the size of the carbonated apatite crystals along the c-axis is inversely proportional to its hardness (7). Literature reports that an increased Ti concentration in hydroxylapatite results in decreased enamel crystal domain size as well as decreased cell lattice parameters (8).

Some extrinsic factors might influence element concentration in dental enamel. Hydrogen peroxide, used for dental bleaching, affects phosphate and carbonate concentration (9-15), however the effect on Ti, among other trace elements, remains unknown. Since Ti is related to a smaller size of hydroxyapatite crystals and thus increased tooth lightness, it was the aim of our study to analyze the effect of tooth bleaching agents on Ti concentration and its variation among the treatment weeks. Therefore, this paper reports on our progress so far with the study of the trace material titanium by modifying whitening agents and shows how this approach can be applied for studying other trace materials.

## Material and Methods

The present research protocol was approved by the Research Commission of the Dental Faculty at the University of Costa Rica.

Research methodology was described elsewhere (15). Briefly, sixty sound human teeth, extracted for periodontal and/or orthodontic reasons were randomly divided into six experimental groups according to the type of whitening gel ( Table 1). Raman spectra readings were performed always on the same spot, previously marked with two lines on the buccal surface of each tooth. This specific area was also the place where the bleaching agent was applied.

**Table 1:**
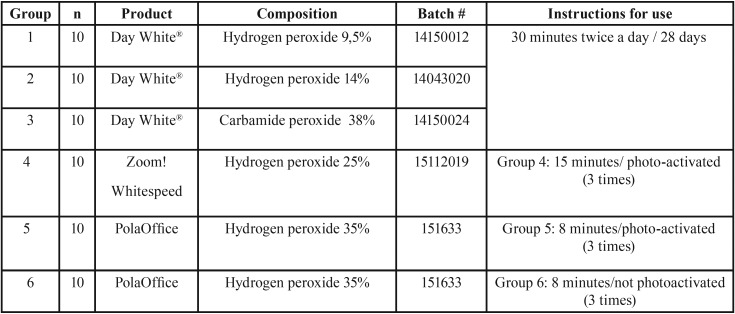
List of treatment groups and materials used.

Measurements were carried out by means of a confocal Raman spectrometer (Model Alpha300 R from the company WiTec, Ulm, Germany), fitted with a 100 mW He/Ne laser (785 nm wavelength). Laser diameter was circa 50 μm. All spectra were measured with an integration time of 0,5 s and one hundred acquisitions. The above in order to enhance the signal-to-noise ratio of the relevant peaks. A typical Raman spectra of dental enamel would have a peak at 139-154 cm-1 correspondent to symmetric titanium dioxide stretching vibration states in all Raman spectra of the dental enamel specimens.

After the base-line readings, bleaching gels were applied and readings were made again at the second and fourth treatment weeks for groups 1-3. Readings for groups 4-6 were made after the first and second bleaching. For the in-office bleaching groups, two consecutive applications were made at the first session, and one application was performed at the second session a week after. Spectra were improved by subtracting the ambient noise from the laser spectra. We also analyzed the intensity of the background in pursuance of the loss of organic content in the teeth after the whitening agent, nevertheless the signal-to-noise ratio was not high enough in order to be able to draw conclusions.

PeakFit® (Systat Software Inc.) software was employed to calculate the area under the curve corresponding to the Ti peak. All data was normalized with the Rayleigh peaks prior to the statistical analysis in order to compensate for the possible variations in the ambient conditions.

Repeated measures analysis of variance (ANOVA) and post hoc Bonferroni test was applied to observe differences among the treatment weeks in each experimental group.

## Results

 Table 2 shows the multivariate analysis for titanium molecules, which change significantly along the treatment weeks, and behave also significantly different among the treatment groups. However, when the experimental groups are analyzed by the type of whitening procedure, at-home versus in-office bleaching, it can be seen that there is no difference among the experimental groups, but rather only among the treatment weeks.

**Table 2:**
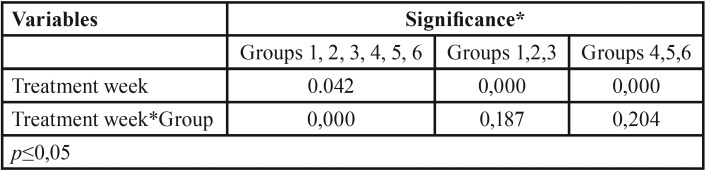
Multivariate test for experimental group and treatment week.

**Figure 1:**
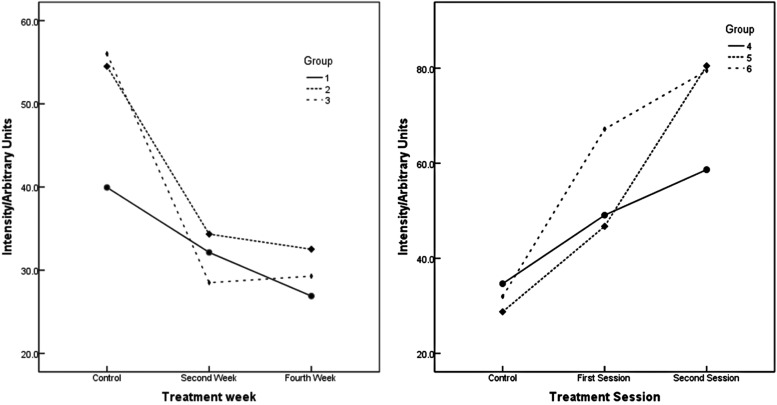
Variation of the Titanium during the treatment weeks.

Figure 1 shows that at-home bleaching agents (groups 1-3) produce a decrease in the titanium molecule. For groups 2 and 3 this decrease was significant at the second and fourth week of treatment, when compared to the respective control week ( Table 3).

**Table 3:**
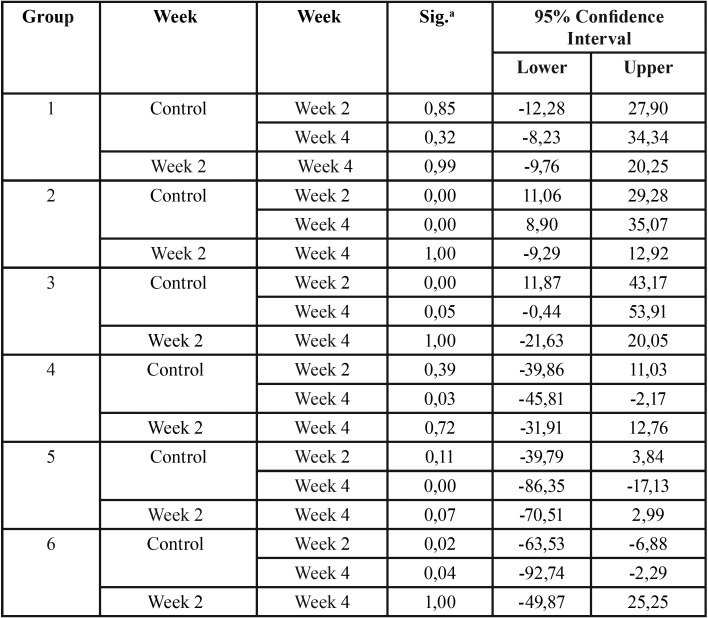
Significant Bonferroni pairwise comparisons.

In-office bleaching agents (groups 4-6) on the other hand, showed a significant increase in Ti ( Table 3) in relation to the control week.

## Discussion

Titanium dioxide is a little studied trace element in dental enamel. However, it is a broadly employed additive in food and pharmaceuticals. Its primary use is as a pigment, because of its brightness and high refractive index (16). A group of researchers analyzed different commercially available products and reported that the foods with the highest content of TiO2 were candies, powered drink mixes and chewing gums with a hard shell, on the other hand personal care products like toothpastes and sunscreens contained up to 1%-10% titanium by weight (16). Few of these products list Ti on the packaging and the mean size of the particles is about 100 to 300nm.

TiO2 is also incorporated in tooth pastes (17) due to its effect on enamel and cementum solubility. Although Ti solutions at high concentrations have a strong acidity and might damage the tooth surface, low concentration solutions combined with highly concentrated fluorides decrease the solubility of cementum. Titanium binds to the organic molecules of the dental tissue and in this way fluorides are trapped in the matrix, making the surface less soluble and allowing a slower release of fluorides over time (18). Cementum is more beneficiated by this effect, since it has a greater organic component than enamel.

Due to the broad employment of TiO2, this molecule is easily dispersed into water and air, which can explain its presence as a trace element in dental tissues. During controlled experiments, our study corroborated the presence of this molecule by means of Raman spectroscopy in all dental specimens analyzed. Furthermore, we could observe that in-office whitening products (hydrogen peroxide at higher concentration applied in fewer sessions) produced a different effect on titanium dioxide in comparison to at-home bleaching gels.

Lower concentration whitening agents employed for a prolonged period of time (at-home bleaching) caused a decrease in titanium concentration. This is in agreement with other publications, and might be because to the degradation of the enamel superficial layer, as well as the oxidation in the organic matter after a prolonged exposure to these chemical substances (9,13). It must be noted that the higher decrease was observed at the second week of treatment, and that it stabilized towards the fourth week.

In-office bleaching protocols caused the inverse effect, since TiO2 increased its concentration during both application sessions, regardless of the activation with light. According to the literature, an increase in Ti is related to a decrease in the size of hydroxyapatite crystals (19), which in turn is related to a higher value (6,8). This mechanism might be one of the reasons why in-office whitening produces a higher value already from the first application since, according to Eimar, smaller enamel crystals cause a higher light scattering. This improvement must be seen with caution, since smaller crystal size is correlated to lower enamel hardness (7).

## Conclusions

The analysis of all dental specimens revealed the presence of titanium dioxide.

The effect of dental bleaching seems to be dependent on the peroxide concentration and on the period of application.

Bleaching gels at higher concentrations and employed on fewer sessions seem to increase the titanium dioxide concentration, whereas gels at lower concentrations employed for a prolonged period of time cause a decrease in the molecule.
